# Health equity related challenges and experiences during the rapid implementation of virtual care during COVID-19: a multiple case study

**DOI:** 10.1186/s12939-023-01849-y

**Published:** 2023-03-11

**Authors:** Simone Shahid, Sophie Hogeveen, Philina Sky, Shivani Chandra, Suman Budhwani, Ryan de Silva, R. Sacha Bhatia, Emily Seto, James Shaw

**Affiliations:** 1 Women’s College Hospital Institute for Health System Solutions and Virtual Care, 76 Grenville Street, Toronto, ON M5S 1B3 Canada; 2 Department of Health Research Methods, Evidence, and Impact, McMaster University, 1280 Main St W, Hamilton, ON L8S 4L8 Canada; 3 Waasegiizhig Nanaandawe’iyewigamig, PO Box 320, Keewatin, ON P0X 1C0 Canada; 4 Peter Munk Cardiac Centre, University Health Network, 585 University Ave, Toronto, ON M5G 2N2 Canada; 5 Institute of Health Policy, Management and Evaluation, Dalla Lana School of Public Health, University of Toronto, 55 College St, Toronto, ON M5T 3M6 Canada; 6 Centre for Digital Therapeutics, Techna Institute, University Health Network, 90 Elizabeth St, Toronto, ON M5G 2C4 Canada; 7 Department of Physical Therapy, University of Toronto, 160-500 University Ave, Toronto, ON M5G 1V7 Canada

**Keywords:** Health equity, Virtual care, Structurally marginalized communities, Access to care, Digital health, Health disparities, Telemedicine

## Abstract

**Background:**

Virtual care quickly became of crucial importance to health systems around the world during the COVID-19 pandemic. Despite the potential of virtual care to enhance access for some communities, the scale and pace at which services were virtualized did not leave many organizations with sufficient time and resources to ensure optimal and equitable delivery of care for everyone. The objective of this paper is to outline the experiences of health care organizations rapidly implementing virtual care during the first wave of the COVID-19 pandemic and examine whether and how health equity was considered.

**Methods:**

We used an exploratory, multiple case study approach involving four health and social service organizations providing virtual care services to structurally marginalized communities in the province of Ontario, Canada. We conducted semi-structured qualitative interviews with providers, managers, and patients to understand the challenges experienced by organizations and the strategies put in place to support health equity during the rapid virtualization of care. Thirty-eight interviews were thematically analyzed using rapid analytic techniques.

**Results:**

Organizations experienced challenges related to infrastructure availability, digital health literacy, culturally appropriate approaches, capacity for health equity, and virtual care suitability. Strategies to support health equity included the provision of blended models of care, creation of volunteer and staff support teams, participation in community engagement and outreach, and securement of infrastructure for clients. We put our findings into the context of an existing framework conceptualizing access to health care and expand on what this means for equitable access to virtual care for structurally marginalized communities.

**Conclusion:**

This paper highlights the need to pay greater attention to the role of health equity in virtual care delivery and situate that conversation around existing inequitable structures in the health care system that are perpetuated when delivering care virtually. An equitable and sustainable approach to virtual care delivery will require applying an intersectionality lens on the strategies and solutions needed to address existing inequities in the system.

**Supplementary Information:**

The online version contains supplementary material available at 10.1186/s12939-023-01849-y.

## Background

Virtual care quickly became of crucial importance to health systems around the world during the COVID-19 pandemic, and its increased use is likely to persist in health care delivery following the end of COVID-19 public health measures. In part because many complex issues must be considered when implementing virtual care such as patient privacy, reimbursement models, new workflows, and technology procurement, the challenge of promoting health equity in uses of virtual care is often overlooked [1]. This problem was made worse during the COVID-19 pandemic, where organizations were forced to implement virtual care in rapid time frames with little support [2]. In this paper we examine whether and how health equity was considered by four health and social service organizations while rapidly implementing virtual care during the first wave (January 2020 to July 2020) of the COVID-19 pandemic in Ontario, Canada, and generate insights for strategies to promote health equity in uses of virtual care.

We define virtual care as “any interaction between patients and/or members of their circle of care, occurring remotely, using any forms of communication or information technologies, with the aim of facilitating or maximizing the quality and effectiveness of patient care” [3, 4]. This involves using information and communication technologies to exchange information during the diagnosis, management, and prevention of disease and illness [5]. Used in complement with or in lieu of in-person encounters, virtual care encompasses a wide array of modalities including phone calls, video conferencing, remote monitoring, asynchronous messaging (e.g., email, texting) and the use of a patient portal. Virtual care also goes by a variety of different terms, such as eHealth, telemedicine, teleconsultation, telecare, and telehealth. The idea of leveraging digital health and virtual care technologies to alleviate existing health system issues have been well documented in the literature [6–8]. Digital health technologies have the potential to increase access to and improve quality of care, lead to time and cost savings [6, 9], and contribute to a more efficient health care system [10]. However, despite the potential of virtual care to enhance access for some communities, health informatics and digital health research has documented risks associated with virtual care initiatives pertaining to health equity [1, 10–13]. In some cases, virtual care initiatives have contributed to widening disparities between structurally marginalized communities and more privileged communities, indicating that the former is less likely to benefit from some virtual care initiatives while the latter is more likely to benefit [11, 13, 14].

In Canada, like many countries around the world, health systems do not serve everyone equitably; this is evidenced by the different access to and outcomes of health care experienced by various population groups [15–18]. Inequities in health status can be attributed to many factors, much of which surface as a result of the historical and ongoing manifestations of colonialism, neoliberalism, and white supremacy [15]. Historical practices of the segregation of Indigenous peoples from the larger population, provision of substandard care to racialized communities, and contemporary experiences of prejudice and discrimination due to health care racism have led to lingering feelings of distrust in health care providers and the larger health care system [19–21]. The impact of social and structural processes of marginalization (e.g., white supremacy, settler colonialism, systemic racism, ableism, ageism, etc.) facilitate control over, exploitation of, and harm to disadvantaged groups and individuals [22]. In recognition of the influence of these intersecting systems that confer advantage and disadvantage to particular communities with respect to health care, we refer to communities characterized by relative disadvantage as *structurally marginalized* by health care systems.

Health inequities become evident in relation to virtual care in a variety of ways. For example, access to a connected digital device is essential to engage in virtual care. However, cost-related challenges to acquiring digital devices and the internet exist across many communities including older adults [23, 24] and racialized groups [23, 25, 26]. In remote and rural communities, those who do not experience cost-related challenges might still struggle with infrastructural issues such as limited internet access and connectivity issues [9, 27]. Many communities, including Indigenous communities, contend with a combination of geographic remoteness and inadequate infrastructure and/or resources to engage in virtual visits [9, 23].

Communities also need to have the appropriate skills to engage in virtual care. Digital health literacy involves the knowledge of how to use a specific technology for a health care purpose and the confidence to act on that knowledge in order to participate actively, regularly, and comfortably in virtual appointments [13]. For example, some research shows that older adults who are less educated and live alone struggle more with using digital health technologies compared to other groups [23]. Furthermore, not all technology has incorporated the appropriate design elements to promote ease of access for those with physical, visual, auditory, and cognitive impairments – further contributing to lower levels of virtual care usage among some groups [23, 24].

While the barriers for structurally marginalized communities to access and receive high quality virtual care have been widely documented in the literature, the rapid introduction of widespread virtual care services into mainstream care delivery at the onset of the COVID-19 pandemic was a novel event worldwide. The scale and pace at which services were virtualized did not leave many organizations with sufficient time and resources to ensure optimal and equitable delivery of care for everyone. The strategies that organizations employed to ensure equitable access to virtual care during the pandemic remain unclear. The objective of this paper is to

1. document the challenges in considering health equity during the rapid implementation of virtual care in Ontario, Canada during COVID-19,
2. document the strategies implemented to mitigate challenges, and
3. explore how access was hindered or achieved based on the results.

## Methods

### Study design

This study followed an exploratory, multiple case study approach drawing on the methodological guidance of Yin [28]. Exploratory case studies are used to gain in-depth descriptions of social phenomenon and allow for comparisons across cases. While variations on case study approaches exist, exploratory case studies seek to answer ‘what’ and ‘how’ questions about the phenomenon of interest. In order to situate the position of the researchers in relation to the research project, we are a diverse group of authors committed to acknowledging and countering systemic inequities embedded in our health care system. We are approaching this work from the perspective of how organizations have responded to rapid changes in care delivery with the intent of informing changes to more equitable health care. For more on our positionality, please see the authors’ information.

### Study setting

We completed exploratory case studies of health and social service organizations that rapidly implemented virtual care during the initial onset of the COVID-19 pandemic in Canada across multiple care settings in Ontario (Table 1). We defined our case studies as a health or social care program, identified by one or more health or social care providers delivering a specific service to a particular population of patients or clients, that had historically been delivered via face-to-face visits and had been rendered virtual in response to the COVID-19 pandemic. We used a maximal variation sampling strategy to recruit four cases across the continuum of care that had varying population densities, including urban hospital-based mental health services, rural community support services, urban home care, and rural primary care. A maximum variation sampling approach enabled us to document the unique features of each case as well as the shared patterns across cases.

**Table 1:** Description of case study organizations

Case	Description	Communities Served
Hospital-based Mental Health Services	An acute care hospital located in an urban centre in Southern Ontario, providing outpatient mental health services by phone and video visits. The organization of which the mental health clinic was a part had made large investments in virtual care in the year prior to the COVID-19 pandemic, and yet the clinic had not implemented virtual visits on such a large scale in advance of the pandemic. The clinic implemented virtual care rapidly with the onset of the pandemic.	Adults requiring mental health services
Community Support Services	Community support services (e.g., meal delivery, caregiver respite, social stimulation activities, etc) offered to multiple townships in rural Ontario. Staff implemented a standardized surveillance instrument conducted over the phone to identify and triage at-risk clients and connect them to the appropriate health or social care service. They implemented a teleconferencing service for patients due to an initial lack of internet accessibility in the office, then introduced videoconferencing shortly afterwards. The volume of patients using virtual care decreased during summer months of 2020.	Rural communities and older adults
Home Care	A home care agency offering a wide variety of home healthcare services in urban centres across Southern Ontario (e.g., physiotherapy, occupational therapy, speech language pathology, nursing, etc). Phone and video visits were authorized for clinical needs related to wellness and health checks, monitoring of conditions/symptoms, remote clinical consultation or intervention related to client care plan goals and support for assessment and reassessment of treatment plans. A virtual care strategy was implemented across all regions within the first 2–3 weeks of the COVID-19 pandemic (March–April 2020). There was an initial dip in the total volume of patients, followed by a return to normal volumes when services were virtualized.	Older adults, individuals with low income, and people experiencing homelessness
Primary Care	Primary care services offered through an Aboriginal Health Access Centre located in Northern Ontario. Services were virtualized primarily through telephone visits and were provided to neighbouring First Nations communities. Providers quickly transitioned to conducting virtual visits from their home. After some feedback about their approach, providers then switched to conducting virtual visits from the community clinics located in First Nations communities. They resumed in-person visits in June 2020 and majority of visits switched back to in-person visits.	First Nations communities** ** Indigenous Peoples in Canada are comprised of First Nations, Inuit, and Métis. This case study included members of some First Nations communities in Northern Ontario.

Table 1 provides an overview of the case studies in this project, including a description of their services and communities served.

### Participant recruitment

We recruited participants through a single study contact (gatekeeper) at each participating organization. Gatekeepers were asked to consider patients who were scheduled for appointments in the past three months. Client participants were included if they were high users of virtual care (those who accepted and used the technology frequently without any problems) and low users (those who struggled with the technology regardless of frequency of use, including those who have declined a virtual visit or those who chose a phone visit over a video visit). Clients were further included to reflect a diverse sample that could offer a variety of perspectives across different characteristics, including multiple gender identities (provide representation of men and women in each case and those who identify outside of the gender identities of man or woman), age (those over the age of 65 and those under the age of 65), and diverse racial and cultural groups (a member of a racialized community, identified by being a person of colour). We also recruited organizational leaders, managers, and providers who were employed at one of the four organizations. A maximum variation sampling approach permitted us to recruit participants with a diversity of perspectives across different identity characteristics such as age, education level, income, and geography (Table 2).

**Table 2:** Participant demographic characteristics

	Care Context			
Characteristics	Primary Care	Home Care	Community Support Services	Hospital-based Mental Health Services
Participant Type				
Patient	4 (35.4%)	3 (30.0%)	3 (33.3%)	3 (33.3%)
Health Care Provider	3 (27.3%)	3 (30.0%)	5 (55.6%)	4 (44.4%)
Manager	3 (27.3%)	3 (30.0%)		
Organizational Leader	1 (9.1%)	1 (10.0%)	1 (11.1%)	2 (22.2%)
Age				
0–55	8 (72.7%)	6 (60.0%)	6 (66.7%)	2 (22.2%)
56+	1 (9.1%)	4 (40.0%)	3 (33.3%)	1 (11.1%)
Unknown	2 (18.2%)			6 (66.7%)
Gender				
Male	3 (27.3%)	2 (20.0%)	1 (11.1%)	1 (11.1%)
Female	7 (63.6%)	8 (80.0%)	8 (88.9%)	2 (22.2%)
Unknown	1 (9.1%)			6 (66.7%)
Racial Group				
White	3 (27.3%)	8 (80.0%)	8 (88.9%)	3 (33.3%)
Indigenous	6 (52.6%)			
Mixed or other	1 (9.1%)	1 (10.0%)	1 (1.1%)	
Unknown	1 (9.1%)	1 (10.0%)		6 (66.7%)
Education Level				
High School				
College	3 (27.3%)	1 (10.0%)	6 (66.7%)	2 (22.2%)
Undergraduate	4 (36.4%)	4 (40.0%)	1 (1.1%)	
Masters	1 (9.1%)	5 (50.0%)	1 (1.1%)	1 (11.1%)
Professional	1 (9.1%)			
Unknown	2 (18.2%)			6 (66.7%)
Geographic Area				
Rural (less 1000 people)	2 (18.2%)		4 (44.4%)	
Small (1000 to 29,999)	8 (72.7)		5 (55.6%)	1 (11.1%)
Medium (30,000 to 99,999)				
Large (100,000 to 999,999)		7 (70.0%)		2 (22.2%)
Urban Centre (1 million+)		2 (20.0%)		
Unknown	1 (9.10%)	1 (10.0%)		6 (66.7%)

Table 2 provides an overview of the sociodemographic characteristics of all participants who partook in the qualitative interviews.

### Data collection

We conducted semi-structured qualitative interviews with participants from all four case studies during the second phase (July 2020 to March 2021) of the COVID-19 Pandemic in Canada. The interview guide was designed in order to accurately convey the experiences of organizational leaders, managers, providers, and patients and included questions in these four categories: 1) comfort level and competency, 2) training supports 3) challenges with virtual care, and 4) benefits of virtual care. We employed a snowball sampling technique, enabling participants to identify other potential participants whose experiences would provide crucial insights to our project. Participants were recruited and interviewed until we reached saturation. Over the course of four months, we conducted 39 interviews.

We conducted approximately 10 qualitative interviews per case. All interviews were conducted by phone (*n* = 12) or by videoconference (*n* = 27). Interview data were collected through audio recordings and detailed note taking. Additional data were acquired through secondary Internet searching for information about the service and about the organizational structure.

### Data analysis

We engaged in rapid analytic methods in order to analyze the interview data on a short timeframe such that our results could be used by case study organizations to improve their virtual services. Rapid evaluation methods have been well documented in the literature [29–34]. Our rapid analytic methods were based on existing best practices in the field [29, 30], and involved summarizing and thematically analyzing the interview data for each case. Two to three members of the research team worked collaboratively to generate domains, themes, and subthemes for each case. Individual themes were identified for each case and used to create a codebook. Interview data were mapped onto the case-specific codebooks and categorized under the themes and subthemes. Research team members met regularly throughout this process to review results and make iterations onto the codebooks. Conflicts arising from divergent data interpretations were resolved through group discussion. A cross-case analysis was subsequently employed to identify differences and similarities across cases and generate high level themes.

## Results

The themes below provide a narrative synthesis of our case studies and outline the challenges experienced by organizations during the rapid implementation of virtual care and the strategies that were implemented to support health equity.

### Challenges

We grouped the challenges identified in our case studies into five distinct categories: (1) Suitability of Virtual Care, (2) Infrastructure for Virtual Care, (3) Digital Health Literacy, (4) Organizational Capacity for Health Equity, and (5) Language and Cultural Appropriateness. Each of the categories produces a distinct form of challenge for health care providers and clients intending to engage in virtual care. Additional file 1: Table S1 details the challenges experienced by organizations offering virtualized services to structurally marginalized communities, and provides supporting quotations from our qualitative data.

Suitability of virtual care relates primarily to whether providers and clients believed virtual care to be a medium through which clinical benefit can be achieved. Many providers were concerned about the usability, effectiveness, and quality of virtual care and believed in-person encounters were more conducive to building better patient-provider relationships. Some providers were so averse to virtual care, they did not even try it; some made a few attempts to engage in virtual care and found it was not suitable for their line of work; and others found that it was appropriate in some instances and should act as a complement to in-person care in the future. The clinical purpose of the virtual visit was essential in determining whether virtual care was an appropriate, or even feasible, mode of care delivery. Appointments requiring hands-on activities, physical tests, and assessments of the home environment were difficult to conduct through virtual modalities.

Infrastructure for virtual care relates to the various material devices and network connections necessary to engage in meaningful virtual care. Service users in our case studies reported experiencing a lack of access to and affordability constraints of the Internet, cellular service, and/or digital devices with sufficient minutes or data. The lack of access was most apparent for those living in rural and remote communities and those who are low-income, including older adults on a fixed income and individuals experiencing homelessness. Additionally, connectivity issues were not limited to patients, and many health care providers also struggled to connect to the Internet for virtual appointments.

Digital health literacy relates to the skills and preparedness to engage in health-related activities in virtual environments, including the telephone. While organizations noted that older adults and individuals who did not speak fluent English experienced the greatest challenge with using technology, even the most technologically savvy patients were described as having difficulties. The low levels of digital health literacy among patients increased the administrative burden for staff and providers who were often left to guide patients through the set-up process of engaging in a video visit. This left less time for the appointment itself, prompting patients and providers to choose phone modalities over video modalities to avoid further impediments to care. Additionally, some of the aversion providers felt about switching to a virtual medium was due to their own lack of digital health literacy. Some providers struggled with learning new software and were not confident in their ability to navigate the technology with enough expertise to provide sufficient and appropriate care.

Organizational capacity for health equity relates to the existing skills, knowledge, and attitudes at an organization regarding the causes of health inequities and strategies to address them. Knowledge about health equity and the role it plays in determining patient access to care was variable among leaders and providers at the organizations. Health equity was not largely considered during the initial implementation process but underwent deeper consideration as virtual care implementation continued. Furthermore, the capacity of organizations to ensure a robust and equitable approach to virtual care delivery was somewhat diminished by their preoccupation with the COVID-19 pandemic. Services were rapidly virtualized during a time with many competing priorities for health care organizations and with ongoing changes with human health resources (e.g., staffing shortages, temporary layoffs, redeployment). Organizations were first and foremost concerned with setting up a virtual care strategy at their respective organizations and began to direct their attention towards reaching specific communities when barriers to access became apparent.

Language and cultural appropriateness relate to the linguistic and cultural meanings of virtual care encounters for clients of varying cultural identities. In some cases, language created immediate and direct barriers because clients did not communicate in English as providers expected. These visits often resulted in interrupted care or shortened visits. Some organizations are currently in the process of developing clinical interpreter processes for virtual appointments, but such processes have yet to be widely implemented. In other cases, cultural beliefs about wellness and care meant that a lack of in-person presence detracted from the potential for healing encounters.

While these categories of challenges transpired across all cases, the primary care case study stands out as a unique case. Indigenous perspectives of health and well-being differ from Western conceptualizations, and we acknowledge that this particular case requires separate treatment and attention in our analysis. The history of settler colonialism in Canada means that members of First Nations communities experienced challenges in a way other participants did not. This, in turn, means the primary care organization needed to consider the diverse needs of their patients in order to respond to these distinct challenges. Table ﻿3 details the challenges experienced by First Nations communities engaging with virtual primary care services.

**Table 3:** Experiences in virtual care for first nations communities

Indigenous cultural safety was an essential component in the primary care case study. Elders and older adults living in the First Nations communities served by the primary care organization were identified as experiencing important challenges with virtual care, especially if they were not fluent in English. Members of the First Nations communities participating in the case study described themselves as being a part of a very *visual culture*. In this sense, visual culture refers to the importance of seeing as a way of knowing the world, and through visions and dreams, represents connection to the spiritual realm. Members of this community described themselves as visual learners, for whom knowledge is acquired through real-life, practical, and hands-on experiences. Beyond the importance of a visual culture, care was not understood by participants in the First Nations community as “transactional”, but rather as *relational*. Care and healing were understood to occur through co-presence, not through the exchange of diagnoses and advice. Connected to these understandings, in-person care was valued for its visual and relational presence because of the energy people bring to one another when they interact. Energy helps with the healing process, but with the rapid onset and widespread implementation of virtual care, First Nations communities had to adapt to offering their energies in a new and different way.	
The rapid switch to virtual care as a result of the pandemic was interpreted by some members of the communities involved to mean that the delivery of health care services had halted, and providers did not want to see them. These beliefs were reinforced by the lived experiences of community members who had experienced health care racism and had been recipients of substandard care from a health care system that has historically refused to treat them. Community clinics had closed in March 2020, during the first wave of the COVID-19 pandemic, and due to the lack of effective communication about the switch to virtualized services, many patients did not realize there were alternative care options available for them. As a result, many issues that could have been resolved virtually went unaddressed and some patients were described to experience poorer health.	
Initially, there was no plan in place to build the self-efficacy of First Nations communities to engage with virtual care in ways that reflect their culture. In order to implement such a large-scale change, the primary care organization required a more fulsome and repetitive communication strategy to inform clients about the switch to virtual care and increase awareness about the available digital health options being provided. In response to feedback from community members, the primary care organization spent a lot of time engaging with community members and leaders of the neighbouring First Nations communities. In general, the organization took its direction from the community leadership when it came to service provision, which now included virtual care. Having incorporated initial feedback about the challenges of virtual care, the organization sought out additional feedback and adapted their services to align with the needs and wishes of communities. An important example was that health care providers began conducting virtual visits from community clinics located in First Nations communities in order to demonstrate their commitment to being present in communities. The clinics were not necessarily open for community members to access, but the physical presence of providers in their local communities reflected the understanding by the organization of the cultural significance of having providers in close proximity to their clients.	

### Strategies

We grouped the strategies identified in our case studies into three distinct categories: (1) Blended Models of Care Provision, (2) Volunteer and Staff Support for Outreach and Virtual Care, and (3) Securing Virtual Care Infrastructure. Additional file 2: Table S2 details the strategies used by organizations offering virtualized services to structurally marginalized communities and offers illustrative quotations.

Blended models of care provision represent the observation that the organizations we studied did not rely exclusively on any one modality of care delivery. Although focus shifted substantially to providing virtual care as a result of the pandemic, in-person visits were still offered for those who were unable to access care any other way, such as those who encountered infrastructural barriers to accessing virtual care, had a health care concern that was considered “essential”, or were comfortable with face-to-face encounters during COVID-19. This commitment to maintaining access was the case across all organizations we studied, and led to the development of an approach in which multiple modalities of care delivery (e.g., virtual visits, home visits, in-person visits, porch visits) were offered to clients in need.

Volunteer and staff support for outreach and virtual care refers to the establishment of processes by which volunteers, staff, or care providers at organizations mobilized to provide necessary education, support, and outreach to enable clients to use virtual care. In many cases, this involved training sessions for clients the day prior to health care visits occurring virtually or a dedicated team of staff available for live support during virtual appointments. When human resources were not available to accomplish this, organizations relied on informal supports in the home or provided a central site (e.g., at the clinic itself) where necessary technology and support were made available. In some cases, staff members went in person to clients’ homes to ensure they had the technology necessary to engage in future virtual care visits.

Securing virtual care infrastructure refers to the systematic efforts of organizations oriented toward ensuring that clients had the necessary network connections and digital devices to engage in virtual care. Many organizations developed or partnered with programs that made digital devices available for groups who did not have access to them. In some instances, devices and equipment were purposefully chosen to suit the needs of specific individuals. For example, Chromebooks were chosen for patients requiring larger screens due to visual difficulties or those who required a self-standing device due to physical impairments; and headphones were distributed to those with auditory impairments. These programs were used to identify those who required the most support to access virtual care including individuals experiencing homelessness, and those who absolutely needed to continue to receive care.

## Discussion

We conducted an exploratory, multiple case study of four organizations providing health and social care services that span the continuum of care in Ontario, Canada. We presented a narrative cross-case synthesis that outlined the challenges organizations experienced during the rapid implementation of virtual care during the first wave of COVID-19 and the strategies they put into place to promote health equity. In this discussion section, we address the issue of access to virtual care for structurally marginalized communities by drawing on a leading framework for conceptualizing access to care, and outline implications for policy and practice in this field.

**Fig. 1:**
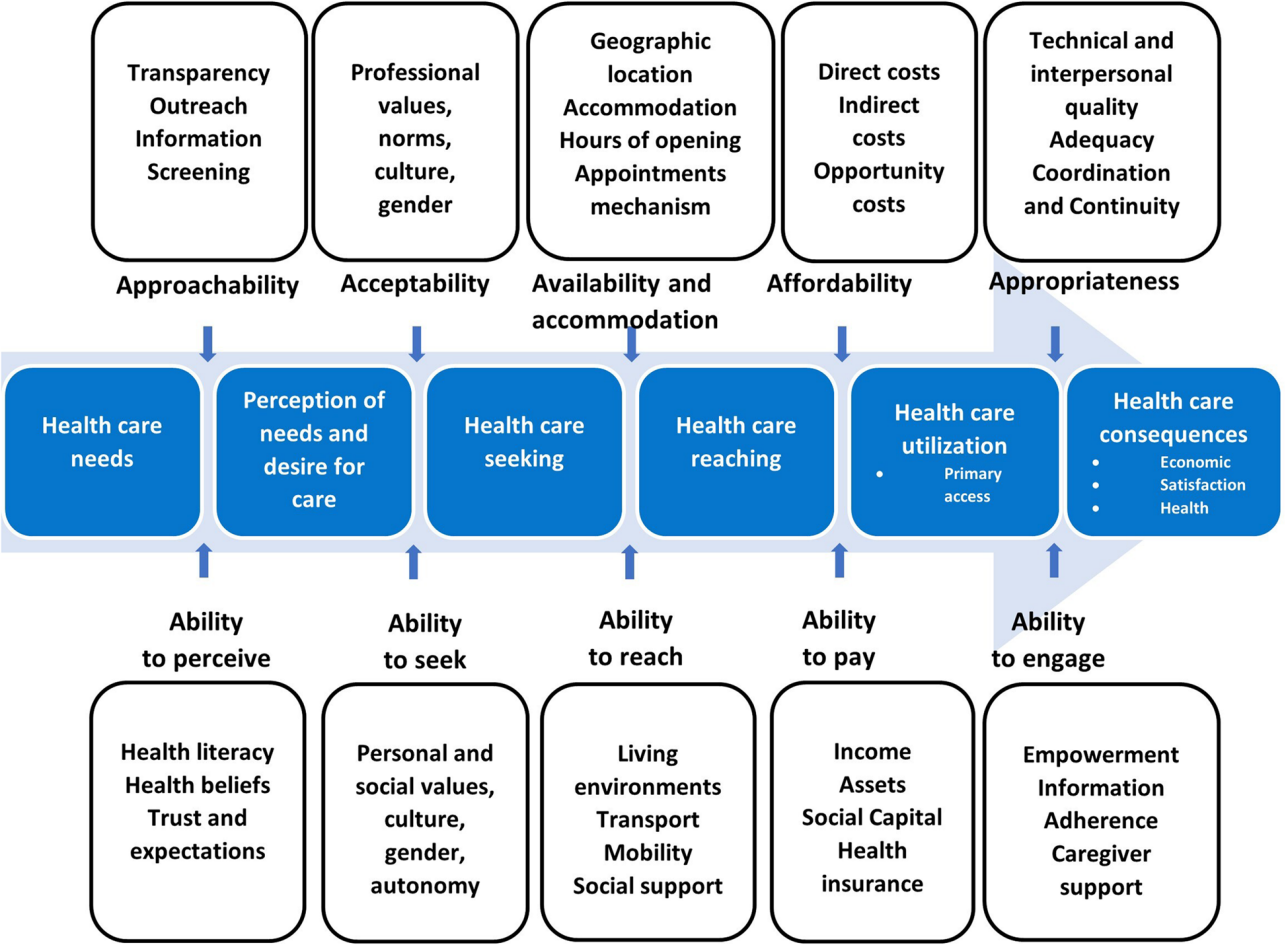
Levesque et al’s [35] conceptual framework of access to health care

Levesque et al. [35] reviewed definitions and conceptual discussions of the concept of access in published literature to produce a comprehensive framework for understanding and studying access to health care (Fig. 1). Attending to both the “supply side”, or features of health care providers and services, and the “demand side”, or features of the individuals and communities seeking out care, they outlined five dimensions of accessibility of health care services: (1) Approachability (2); Acceptability (3); Availability and accommodation (4); Affordability; and (5) Appropriateness. Each of these dimensions of accessibility corresponds to an ability of individuals or communities to realize access to care, requiring a set of structural, social, and geographic circumstances that enable interaction with health services along the process of seeking care and benefiting from services. In this way, they suggest that “access is seen as resulting from the interface between the characteristics of persons, households, social and physical environments and the characteristics of health systems, organisations and providers”. Our multiple case study has focused on the actions of health care organizations to promote access to virtual care during the early phases of the COVID-19 pandemic, and as such, we structure our discussion according to the actions of organizations in each dimension of accessibility. However, the accessibility and ability dimensions are not independent constructs but are interconnected and influence one another. We acknowledge that the ability dimensions are not solely the responsibility of the patient but rather are a reflection of how systems are designed to respond in a way that facilitates or impedes patients to act on those abilities.

### Approachability

Approachability refers to the possibility that people searching for services can “actually identify that some form of services exists, can be reached, and have an impact on the health of the individual”. At the immediate onset of the COVID-19 pandemic, there was a significant drop in utilization of health care in Canada [36]. However, that quickly recovered as patients and providers engaged with virtual care. Generally speaking, people across our case studies were aware that virtual care was available as a result of the prominence of the impacts of the pandemic on health care in news media and social media. When people contacted the organizations in our study to seek out health care, they were presented with a set of options aligning with the rapid shift to virtual care across organizations. In some cases, patients cancelled appointments or chose not to create appointments that were virtual, however, the widely publicized nature of the shift to virtual care meant that, apart from the *primary care case* described earlier, approachability was not a primary barrier to access in our dataset.

Nonetheless, organizations in our study eventually sought to enhance the approachability of virtual services as the pandemic evolved. The strategy “*volunteer and staff support for outreach and virtual care*” included specific activities such as community engagement to educate communities about available virtual services and gain feedback about what efforts are required to make virtual care more appealing and satisfactory. Although beneficial, questions were raised about the sustainability of volunteer and staff approaches to community engagement and education in the longer term. The literature pointed to additional strategies that did not appear in our case studies, including educating the larger community to raise awareness about virtual care initiatives [23, 24, 37], involving the community during the implementation process [37], gradually introducing the virtual care technology [23], and deeply engaging in community consultation to seek input about users’ needs [38]. We propose that investing in health equity in virtual care for the future will need to more fully engage with these approaches to enhancing the approachability of virtual care.

### Acceptability

Acceptability refers to the cultural and social influences on whether people accept aspects of the service. The clearest and most obvious challenges that interfered with access to virtual care under the acceptability domain is the challenge “*language and cultural appropriateness”* especially as it relates to approaches to care. As detailed in Table ﻿3, many members of the First Nations community included in our study did not feel that virtual care was implemented in a way that resonated with their cultural beliefs about health, wellness, and healing. A distinct but related issue pertains to the language spoken during medical appointments, where patients were less able or unable to communicate with health care providers virtually as a result of low fluency in English. These two examples were obvious barriers to virtual access to care, and will require investment in explicit strategies to address them for sustainable futures of virtual care.

Organizations in our study employed strategies related to acceptability in the “*volunteer and staff support for outreach and virtual care*” category, as well as the “*blended models of care provision*” category. With respect to the First Nations community described earlier, a blended model of care involving the community presence of providers, in-person visits where necessary, and virtual visits where acceptable substantially enhanced the acceptability of care. Several other strategies have been documented in the literature, which organizations can consider implementing, to enhance the acceptability of virtual care. Some of these strategies include language and culture matching to facilitate communication [26, 39, 40], and ensuring interpretive services [41, 42] and translated materials [42, 43] are available and accessible. Other strategies involve designing and providing culturally appropriate technology [9, 26] and services [9, 25, 44], and training providers to deliver care in culturally appropriate ways [26, 39, 43–45]. This may involve incorporating elements of spirituality and traditional medicine, considering holistic health, and incorporating cultural norms and local beliefs.

### Availability and accommodation

Availability and accommodation refer to the existence of resources within a health care system that are of sufficient quantity and quality to produce and deliver services. In this way, availability and accommodation relate to when, where and through which modality health care services are offered as well as the characteristics of who is offering the services, and whether these features align with the needs and wishes of people pursuing care. Barriers to access documented in our study linked to a number of dimensions of availability and accommodation. For example, where staff experienced concerns with their own “*digital health literacy*”, they were less able to seamlessly engage patients in virtual visits. However, the most salient challenge in this dimension of access relates to low levels of “*organizational capacity for health equity*”, such that organizations lacked deep expertise in the causes and consequences of inequities in care. This is a particularly salient point related to equitable access to virtual care.

Although organizations promoted access in the availability and accommodation dimension via strategies in the “*blended models of care provision*” category and eventually through the “*volunteer and staff support for outreach and virtual care*” categroy, the challenge of acknowledging the broader social, structural, and political determinants of the health system structure and access to care was prominent. Most participants were not explicitly aware of these broader challenges or knowledgeable about the ways they impact the availability and accommodation of access to care. Interview discussion rarely approached dialogue about the underlying determinants that generate inequities and influence access to care, such as income inequality, settler colonialism, and systemic racism. Acknowledging these contexts in which virtual care is implemented is a crucial first step in promoting health equity in virtual care. Such acknowledgement will facilitate deeper awareness about the particular kinds of accommodation that are required, and inspire deeper engagement in learning about the causes and consequences of health inequities in systems of care.

While virtual care does not address fundamental issues related to transportation, provider shortages, and insufficient health care services in Indigenous and rural communities, it does have the potential to enhance access by facilitating connections to remote providers and reducing travel time and transportation costs [9, 27, 39]. The absence of adequate infrastructure and providers in these communities belies the continued lack of prioritization of Indigenous health and exemplifies the inequitable and inadequate distribution of resources across regions, organizations, and communities.

### Affordability

Affordability refers to the “economic capacity for people to spend resources and time to use appropriate services”. Importantly, affordability is not solely about the possible direct costs of care that might be covered via public taxation, private insurance, or out-of-pocket spending, but also the indirect costs associated with time away from work, the need to travel, or in the case of virtual care, the need for digital technology and high-speed Internet access. Indeed, the lack of access to and challenges associated with these latter elements of the “*infrastructure of virtual care*” category was an important finding in our study. This outcome is supported by existing literature detailing the lack of affordability and access to digital devices and/or the Internet among structurally marginalized communities, such as racial and cultural minorities [25, 46], older adults [23, 24, 47], Indigenous Peoples [9, 38, 43], individuals living in rural and remote settings [9, 38], and individuals with low income [46].

Organizations in our study leveraged strategies related to the “*blended models of care*” (e.g., offering phone visits where patients did not have access to Internet-connected digital devices) category. Blended models of care represent a crucial set of strategies that are necessary to promote access in the affordability dimension and facilitate access for patients in all financial circumstances. Another promising strategy to promote access to virtual care specifically involves “*securing virtual care infrastructure*” for patients. For example, donating digital devices where necessary is a promising strategy, documented both in the case studies and in the literature [24, 26, 38, 48]. Furthermore, systematic reviews by Bradford et al. [49] and Kruse et al. [9] recommend using low-cost alternatives to more expensive equipment, while Fang et al. [23] recommends creating social policies introducing subsidies to purchase a digital device for individuals with low income, and further facilitating access to virtual care by ensuring that the appropriate technology is available in easily accessible spaces. Where possible, organizations should advocate for subsidies as opposed to lost-cost alternatives, which may have an implication on the quality of the equipment and services being offered. These additional strategies will promote a more comprehensive approach to ensuring affordability for future uses of virtual care.

### Appropriateness

Appropriateness refers to the fit between the person’s needs, the nature of the services offered, and the timeliness of care. Appropriateness is thus about whether the services are of sufficient quality and type to actually meet peoples’ needs. Organizations in our study expressed concerns about the appropriateness or “*suitability of virtual care*”, especially with regards to the modality being offered (e.g.whether a telephone visit was sufficient to address complex health issues). Challenges related to appropriateness have also been documented in the literature, including low levels of digital health literacy [9, 25, 46, 50, 51] and a mismatch between the technology used and a patient’s sensory, physical, and cognitive ability [24, 52–56].

Organizations in our study produced clearly stated workflows and enhanced health care provider training to address these issues. For example, organizations offered education and practice sessions to patients who uncomfortable with accessing virtual care. By setting up appointments to review the process of a virtual visit and answer patients’ questions, organizations enabled those with lower digital literacy to view a digitally-mediated health care visit as acceptable. This strategy points to the demand for widespread digital health literacy training as a strategy to promote the equitable and ongoing use of virtual care. However, important gaps related to the fit between the modality being offered and the capacity and needs of patients remained. Appropriateness of virtual care is multi-dimensional, relating to digital health literacy, the availability of education and support, and the nature of the clinical or social need being addressed. Future effort to promote the appropriateness of virtual care will require this multi-dimensional perspective.

#### Overarching reflections and implications for health systems

Although barriers to access for structurally marginalized communities and their underlying causes are well documented in the literature, health equity was not a universal priority concern during the rapid virtualization of care. The unexpected crisis and the rush of the pandemic left little time for health care organizations to implement virtual care in an equitable way, as their priority was to maintain continuity of care. This strategy may have inadvertently contributed to increasing inequities within the most structurally marginalized communities.

Our research project has illustrated a number of important themes related to the infrastructure for virtual care, organizational capacity to engage with virtual care in meaningful ways, and existing inequities in the broader health care system. However, even as organizations begin to familiarize themselves with the evidence base and shift their attention towards equitable service delivery, it is apparent that these inequities are persisting and are embedded in inequitable systems. Any effort to develop a plan for the sustainability of virtual care services requires an understanding of the potential consequences for members of structurally marginalized communities who may have precarious access to many virtual care initiatives. A lack of awareness and engagement of these existing inequities simply meant these challenges also manifested in virtual care delivery. Ultimately, individual providers and organizations are unable (and do not have the power) to solve system-level challenges on their own.

As health systems in Canada and elsewhere work towards comprehensively integrating virtual care into care delivery, local and national governments will need to work collaboratively to ensure that an equitable and sustainable approach to virtual care delivery is in place. Our analysis points towards the policy recommendations arising from Budhwani et al. [57], related to recommendations aimed at the individual, technological, health system, and social/structural determinants level. In addition to the strategies outlined above, governments can consider investing in subsidized options for cellular phone service and high-speed Internet to promote equitable access to the connectivity required to engage in virtual visits [2] and commit to ensuring that high-speed Internet and cellular service is made available across the entire geography of a region. Governments, health systems, and health care leaders can also invest in educational content to build capacity in understanding equity, inclusion, diversity, and anti-racism in health care organizations [2]; and to advocate for the systematic and comprehensive inclusion of equity, diversity, and anti-racism education into the formative training of health care providers and managers.

## Limitations

One limitation of this study is the rapid timeline in which it was conducted. The rapid nature of this project was necessary due to the urgency of producing timely results for organizations looking to improve virtual care delivery during COVID-19. Rapid analytic methods are conducive for analyzing many interviews within a shortened timespan and are an efficient and rigorous approach for identifying key implementation characteristics [30] and providing quick and actionable feedback [29, 31–34].

While case study results are often stated to be ungeneralizable to the wider population, many concepts can still be transferrable to other settings [58, 59]. The insights generated from the cases were intended to help create a foundation for understanding the real-world challenges and strategies that characterize efforts to promote health equity in virtual care.

We acknowledge that despite our effort to recruit a diverse sample of participants to interview, many of them identified as older, educated, medium and high income, white women. Given that our only form of contacting patients was through virtual means, it was difficult for our team to reach those with no or infrequent access to virtual technologies. The need to practice physical distancing during the COVID-19 pandemic interfered with the possibility of meeting participants for in-person interviews.

### Directions for future research

Future studies should focus on organizations whose provision of care is primarily delivered to members of structurally marginalized communities and who have expertise in engaging with these communities. Work in this direction should highlight examples from organizations that have successfully implemented strategies that improve access to virtual care for specific structurally marginalized communities. Future work should also explore perspectives from communities who were underrepresented in our project. Subsequent research could be done in a non-rapid context and employ traditional analytic methods. Such methods should also involve deeper and prolonged community engagement, drawing on methodologies such as community-based participatory research (CBPR).

## Conclusion

While the rapid scale-up of virtual service delivery manifested as a timely response to the COVID-19 pandemic, health equity was not a priority concern during this process. During the rapid implementation of care, organizations prioritized provider-friendly processes and missed the mark in providing equitable client-centered care. Organizations did not initially consider or incorporate the pieces required to make virtual care work for the most structurally marginalized individuals and communities. This paper outlined the challenges and strategies experienced by four organizations in Ontario that rapidly virtualized their health care services. Drawing on a framework that conceptualizes access to health care, we examined the key accessibility dimensions that were emphasized and overlooked by organizations.

The inequitable distribution of health care resources arising from persistent colonial systems and practices have far-ranging consequences, and these include their impact on inequitable access to virtual care.

## Supplementary Information

**Additional file 1: Table S1.** Challenges Identified during the Rapid Implementation of Virtual Care. **Table S1.** details the challenges experienced by organizations offering virtualized services to structurally marginalized communities and provides supporting quotations from our qualitative data.

**Additional file 2: Table S2.** Strategies Implemented during the Rapid Implementation of Virtual Care. **Table S2** details the strategies used by organizations offering virtualized services to structurally marginalized communities and offers illustrative quotations.

## Data Availability

Data are not publicly available from the authors for this research project as they were funded by a government entity and are protected by privacy and confidentiality policies.
